# Genetic variation affecting DNA methylation and the human imprinting disorder, Beckwith-Wiedemann syndrome

**DOI:** 10.1186/s13148-018-0546-4

**Published:** 2018-08-30

**Authors:** Vinod Dagar, Wendy Hutchison, Andrea Muscat, Anita Krishnan, David Hoke, Ashley Buckle, Priscillia Siswara, David J. Amor, Jeffrey Mann, Jason Pinner, Alison Colley, Meredith Wilson, Rani Sachdev, George McGillivray, Matthew Edwards, Edwin Kirk, Felicity Collins, Kristi Jones, Juliet Taylor, Ian Hayes, Elizabeth Thompson, Christopher Barnett, Eric Haan, Mary-Louise Freckmann, Anne Turner, Susan White, Ben Kamien, Alan Ma, Fiona Mackenzie, Gareth Baynam, Cathy Kiraly-Borri, Michael Field, Tracey Dudding-Byth, Elizabeth M. Algar

**Affiliations:** 1 Department of Paediatrics, University of Melbourne, Parkville, 3052 Australia; 2 Pathology, Monash Health, Clayton, 3168 Australia; 3 School of Medicine, Deakin University, Geelong, 3216 Australia; 4 Victorian Comprehensive Cancer Centre, Parkville, 3052 Australia; 5 Department of Biochemistry and Molecular Biology, Monash University, Clayton, 3800 Australia; 6 Murdoch Children’s Research Institute, Parkville, 3052 Australia; 7 Department of Anatomy and Developmental Biology, Monash University, Clayton, 3800 Australia; 8 Department of Medical Genomics, Royal Prince Alfred Hospital, Camperdown, 2050 Australia; 9 Clinical Genetics, Liverpool Hospital, Liverpool, 2170 Australia; 10 Clinical Genetics, Children’s Hospital at Westmead, Westmead, 2145 Australia; 11 Centre for Clinical Genetics, Sydney Children’s Hospital, Randwick, 2031 Australia; 12 School of Medicine, University of Western Sydney, Penrith, 2751 Australia; 13 School of Medicine, University of Sydney, Camperdown, 2006 Australia; 14 Auckland District Health Board, Auckland, 1023 New Zealand; 15 South Australian (SA) Clinical Genetics Service, SA Pathology, Women’s and Children’s Hospital, Adelaide, 5000 Australia; 16 School of Medicine, University of Adelaide, Adelaide, 5000 Australia; 17 Department of Clinical Genetics, Royal North Shore Hospital, St Leonards, 2065 Australia; 18 School of Women’s and Children’s Health, University of NSW, Kensington, 2052 Australia; 19 Hunter Genetics, Hunter New England Local Health District, New Lambton, 2305 Australia; 20 Genetics Services of Western Australia, Crawley, 6009 Australia; 21 University of Newcastle GrowUpWell Priority Research Centre, Callaghan, 2308 Australia; 22 Hudson Institute of Medical Research, Clayton, 3168 Australia; 23 Department of Translational Medicine, Monash University, Clayton, 3168 Australia

**Keywords:** DNA methyltransferase 1, Beckwith-Wiedemann syndrome, One-carbon pathway, Methylation

## Abstract

**Background:**

Beckwith-Wiedemann syndrome (BWS) is an imprinting disorder with a population frequency of approximately 1 in 10,000. The most common epigenetic defect in BWS is a loss of methylation (LOM) at the 11p15.5 imprinting centre, KCNQ1OT1 TSS-DMR, and affects 50% of cases. We hypothesised that genetic factors linked to folate metabolism may play a role in BWS predisposition via effects on methylation maintenance at KCNQ1OT1 TSS-DMR.

**Results:**

Single nucleotide variants (SNVs) in the folate pathway affecting methylenetetrahydrofolate reductase (*MTHFR*), methionine synthase reductase (*MTRR*), 5-methyltetrahydrofolate-homocysteine *S*-methyltransferase (MTR), cystathionine beta-synthase (*CBS*) and methionine adenosyltransferase (*MAT1A*) were examined in 55 BWS patients with KCNQ1OT1 TSS-DMR LOM and in 100 unaffected cases. *MTHFR* rs1801133: C>T was more prevalent in BWS with KCNQ1OT1 TSS-DMR LOM (*p* < 0.017); however, the relationship was not significant when the Bonferroni correction for multiple testing was applied (significance, *p* = 0.0036). None of the remaining 13 SNVs were significantly different in the two populations tested. The *DNMT1* locus was screened in 53 BWS cases, and three rare missense variants were identified in each of three patients: rs138841970: C>T, rs150331990: A>G and rs757460628: G>A encoding NP_001124295 p.Arg136Cys, p.His1118Arg and p.Arg1223His, respectively. These variants have population frequencies of less than 1 in 1000 and were absent from 100 control cases. Functional characterization using a hemimethylated DNA trapping assay revealed a reduced methyltransferase activity relative to wild-type DNMT1 for each variant ranging from 40 to 70% reduction in activity.

**Conclusions:**

This study is the first to examine folate pathway genetics in BWS and to identify rare DNMT1 missense variants in affected individuals. Our data suggests that reduced DNMT1 activity could affect maintenance of methylation at KCNQ1OT1 TSS-DMR in some cases of BWS, possibly via a maternal effect in the early embryo. Larger cohort studies are warranted to further interrogate the relationship between impaired MTHFR enzymatic activity attributable to *MTHFR* rs1801133: C>T, dietary folate intake and BWS.

**Electronic supplementary material:**

The online version of this article (10.1186/s13148-018-0546-4) contains supplementary material, which is available to authorized users.

## Background

The human imprinting disorder Beckwith-Wiedemann syndrome (BWS) (OMIM 130650) is characterised by overgrowth in the prenatal and postnatal period, macroglossia, umbilical hernia or exomphalos, neonatal hypoglycaemia, ear lobe creases and pits, nevi flammei, hemihyperplasia and organomegaly, particularly of the kidney, liver and pancreas. One of its more serious complications is an increased predisposition to cancer in early childhood with tumour risk segregating with distinct genetic and epigenetic subtypes (reviewed in [1–4]).

The majority of the affected children have an isolated epigenetic abnormality at the 11p15.5 imprinting centre 2 (IC2), known as KCNQ1OT1 TSS-DMR, the location of the *KCNQ1OT1/CDKN1C* locus. Fifty percent of all children with features of BWS have either mosaic or complete loss of methylation (LOM) within the *KCNQ1OT1* promoter and impaired expression of maternal *CDKN1C*, a critical regulator of growth during early development [5, 6]. For the majority of BWS cases with KCNQ1OT1 TSS-DMR imprinting disruption, the aetiology is unknown and the phenotype arises without any evidence of heritability. Rare cases have been reported with imprinting defects in KCNQ1OT1 TSS-DMR associated with inherited recessive mutations affecting *NLRP2* on 19q13.42 [7], and more recently, maternal effect missense and truncating variants in other NLRP loci, including NLRP5 and NLRP7, were reported in the mothers of offspring with multi-locus imprinting disruption (MLID) that included loss of methylation at KCNQ1OT1 TSS-DMR [8, 9]. MLID cases with methylation loss at KCNQ1OT1 TSS-DMR frequently had characteristics of BWS. Animal studies have shown that defective methylation is set in the gametes or during the first cell divisions of the embryo, and genetic modifiers and environmental factors influence this process [10]. Indeed, in BWS, the increased incidence of BWS cases with KCNQ1OT1 TSS-DMR LOM after assisted reproduction (ART) points to the involvement of environmental factors that perturb methylation at KCNQ1OT1 TSS-DMR or alternatively suggest a link between therapeutic interventions for female infertility and defective methylation [11–15].

The one-carbon pathway is the major metabolic pathway through which dietary folate is converted to methyl donor groups that subsequently methylate DNA via catalysis of the universal methyl donor *S*-adenosyl-methionine in the presence of DNA methyltransferase 1 (DNMT1). The key enzymes of this pathway include methylenetetrahydrofolate reductase (*MTHFR*), methionine synthase reductase (*MTRR*), 5-methyltetrahydrofolate-homocysteine *S*-methyltransferase (MTR), cystathionine beta-synthase (*CBS*) and methionine adenosyltransferase (*MAT1A*), and the associated effects of genetic variants with impaired enzyme activity have been documented in several studies. A common SNV (previously commonly known as c.677C>T) in *MTHFR*, rs1801133: C>T, NP_005948.3: p.Ala222Val, increases protein thermolability [16] and has been linked to vascular thromobosis, hyperhomocysteinaemia and global hypomethylation in a setting of dietary folate depletion ([17–20]. A second *MTHFR* SNV, rs1801131: A>C, NP_005948.3: p.Gln429Ala, is linked to DNA hypomethylation, independently of folate availability and acts synergistically with rs1801133 to reduce MTHFR activity [21, 22]. MTR catalyses the remethylation of homocysteine to methionine and SNV rs1805087: A>G, NP_000245.2: p.Asp919Gly alters the helix structure in the substrate binding domain leading to impaired methionine biosynthesis and reduced methyl donor availability [23–25]. *MTRR* rs1801394: A>G, NP_002445.2 p.Ile22Met, acts synergistically with homozygous *MTHFR* rs1801133: C>T, NP_005948.3: p.Ala222Val, in the presence of low folate and/or vitamin B12 status [26].

The isoforms of Dnmt1 (Dnmt1o and Dnmt1s) play a critical role in the maintenance of DNA methylation at imprinted regions during pre-implantation mammalian development [27]. The absence of 118 N terminal amino acids allows Dnmt1o protein to accumulate to high levels in non-dividing growing oocytes [28], and expression is maintained in pre-implantation embryos in the morula and blastocyst. Howell et al. 2001 showed that *Dnmt1o*, while not essential for imprint establishment in oocytes, was required for the maintenance of methylation at imprinted loci, and heterozygous offspring of *Dnmt1o*-null mothers exhibited imprinting defects. The functions of Dnmt1o and Dnmt1 are interchangeable as Dnmt1 is itself able to maintain methylation during pre-implantation development [29, 30], and Dnmt1o can rescue *Dnmt1*−/− ES cells [31]. However, although these studies implicate DNMT1 in imprinting disorders, it has not previously been examined in the context of BWS or MLID.

In this study, we investigated the genetics of folate metabolism and maintenance of DNA methylation in cases of BWS with loss of methylation at KCNQ1OT1 TSS-DMR. This included an analysis of SNVs in *MTHFR*, *MTRR*, *MTR*, *MAT1A* and *CBS* predicted to alter the function and a full mutation screen of *DNMT1*. While a statistically significant association between deleterious SNVs in folate pathway genes could not be conclusively demonstrated, we identified three patients with rare missense amino acid substitutions in *DNMT1* that are represented in dbSNP at a frequency of < 0.001. Functional characterization of these variants showed a significant reduction in complex formation with hemimethylated DNA, consistent with impaired DNMT1 activity. This is the first study examining the folate pathway and the major human methyltransferase, *DNMT1*, in the most common epigenetic subtype of BWS.

## Results

### Analysis of *MTHFR*, *MTRR*, *MTR*, *MAT1A* and *CBS* in BWS cases with KCNQ1OT1 TSS-DMR LOM

**Fig. 1:**
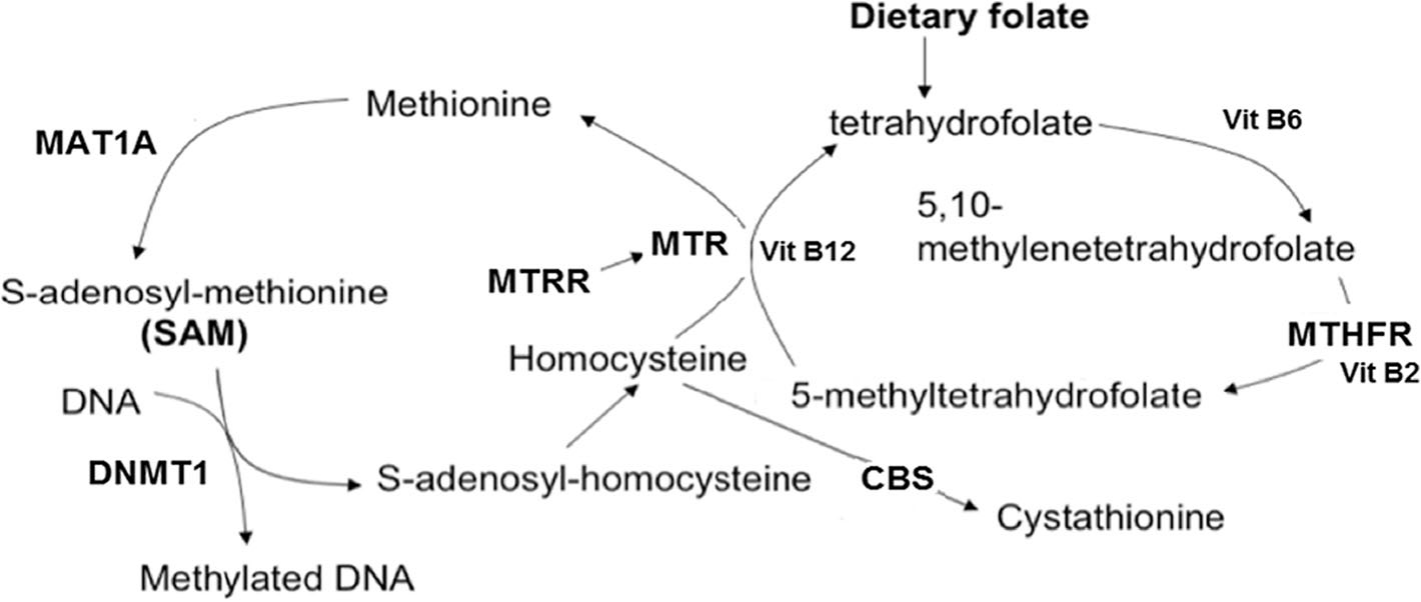
The one-carbon pathway responsible for the conversion of dietary folate to methyl donor groups. The critical enzymes in the pathway examined in this study are shown in bold. MTHFR is primarily responsible for the conversion of tetrahydrofolate to methylene-tetrahydrofolate. MTRR and MTR work together to catalyse the conversion of homocysteine to methionine in the presence of the cofactor vitamin B12 and zinc, enabling the movement of the methyl group from methylene-tetrahydrofolate and recycling of tetrahydrofolate. CBS converts homocysteine in the presence of vitamin B6 to cystathione and MAT1A is responsible for converting methionine to S-adenosy-methionine (SAM), the ultimate methyl donor for DNA via catalysis by DNA methyltransferase

The one-carbon pathway of folate metabolism is shown in Fig. 1. Coding SNVs in *MTHFR*, *MTRR* and *MTR* generating missense amino acid substitutions and for which previous evidence of impaired or altered enzyme function had been demonstrated in other studies were selected for investigation in the BWS study population (55 cases) and in a matched local control population (100 cases). The enzymes MAT1A and CBS were also included because rare coding SNVs were identified in genome databases with damaging polyphen scores and predicted structural effects.

All gene SNVs interrogated in the study are shown in Table 1.

**Table 1:** SNVs interrogated in BWS patients with loss of methylation at KCNQ1OT1 TSS-DMR

Gene	SNV_1 ID	SNV_2 ID	SNV_3 ID	SNV_4 ID
MTHFR	677C>T Rs 1801133: C>T	Rs 1801131: A>C	Rs 2274976: G>A	
	NM_005957.4: c.665C>T	NM_005957.4: c.1286A>C	NM_005957.4:c.1781G>A	
	NP_005948.3 p.Ala222Val	NP_005948.3 p.Gln429Ala	NP_005948.3 p.Arg594Gln	
	A = 0.24	G = 0.24	T = 0.075	
MTRR	Rs 1801394: A>G	Rs 2287780: C>T	Rs 10380: C>T	
	NM_002454.2: c.66A>G	NM_002454.2: c.1243C>T	NM_024010.2: c.1864C>T	
	NP_002445.2 p.Ile22Met	NP_002445.2 p.Arg415Cys	NP_076915.2 p.His622Tyr	
	G = 0.36 GMAF, 0.47 ClinVar, 0.45 EXAC	T = 0.0679	T = 0.219	
MAT1A	Rs 114494303: G>A	Rs 72558181: G>A	Rs 112848063: A>G	Rs 116659053: G>A
	NM_00429.2: c.530G>A	NM_00429.2: c.791G>A	NM_00429.2: c.1061A>G	NM_00429.2: c.1066G>A
	NP_00420.1 p.Arg177Gln	NP_00420.1 p.Arg264His	NP_00420.1 p.Asp354Gly	NP_00420.1 p.Arg356Trp
	A = 0.0002	A = 0.000009		A = 0.0002
MTR	Rs 1805087: A>G			
	NM_000254.2: c.2756A>G,			
	NP_000245.2 p.Asp919Gly			
	G = 0.218			
CBS	Rs 17849313: G>C	Rs 117687681: C>T	Rs 11700812: G>A/C	
	NM_001178009.1: c.205G>C	NM_001178009.1: c.1105C>T	NM_000071.2: c.1106G>A, G>C	
	NP_000062.1 p.Ala69Pro	NP_000062.1 p.Arg369Cys	NP_001171479.1 p.Arg369His,	
		A = 0.0012	NP_001171479.1 p.Arg369Pro.	
			T/G = 0.00003	

Allele frequency data was obtained from dbSNP147 or other sources as indicated. Minor allele nucleotides on the forward genomic strand are shown. Where the alternate allele frequency is not shown, the SNV frequency is unknown. The MTHFR variant commonly referred to as c.677C>T is MTHFR: NM_005957.4 c.665C>T in HGVS format. Nucleotide numbering uses + 1 as the A of the ATG translation initiation codon

Fifty-five unrelated cases with clinical features of BWS were assessed as having either complete or partial LOM at KCNQ1OT1 TSS-DMR. Ten of these 55 cases had multi-locus imprinting disruption (MLID) involving losses of methylation at additional imprinting domains including *MEST*, *PLAGL1*, *GRB10*, *NESPAS* and *PEG3* (Additional file 1: Table S1). A total of 11 cases had been conceived using assisted reproductive technologies (ART). Specimens were screened using HRM across selected one-carbon pathway SNVs, and those with shifted melt profiles were sequenced. Specific reaction conditions are shown in Additional file 1: Tables S2 and S3. The significance of minor allele SNV frequencies in the BWS study population for each gene was calculated using the chi-squared analysis relative to the frequencies determined in 100 control DNA specimens from the cord blood.

*The MTHFR rs1801133 minor allele is more prevalent in BWS with KCNQ1OT1 TSS-DMR LOM*.

Table 2 shows SNV frequencies in BWS patients with KCNQ1OT1 TSS-DMR LOM, including those with MLID, compared to controls and frequencies in dbSNP147. One SNV of 14 screened, *MTHFR* rs1801133: C>T, was significantly different when compared to the local control population with a *p* value of 0.0167 derived using a 2 × 2 contingency table. There were 43 T alleles in a total of 110 in the BWS subgroup compared to 52 T alleles in a total of 200 alleles in the control group. *MAT1A* and *CBS* minor allele frequencies were too low in both populations for meaningful statistical analysis to be done. After correcting for multiple testing of each of the 14 one-carbon pathway SNVs examined, the Bonferroni significance value was 0.0036 (0.05/14). The *p* value for *MTHFR* rs1801133: C>T at 0.0167 was no longer significant when this more stringent statistical test was applied to the data. Hence, although MTHFR rs1801133: C>T appears to be more prevalent in the BWS population, this finding is not statistically significant when adjustment for multiple testing is applied.

**Table 2:** Table showing one carbon pathway enzyme allele frequencies in 55 BWS and 100 control specimens and their significance values

Gene	SNV	BWS MAF 110 alleles	Local control MAF 200 alleles	*p* value BWS versus local controls	Global control MAF
MTHFR	Rs1801133: C>T	0.391	0.260	*0.0167*	0.24
	Rs1801131: A>C	0.309	0.400	0.0516	0.2494
	Rs2274976: G>A	0.045	0.060	0.6606	0.075
MTRR	Rs1801394: A>G	0.560	0.490	0.214	0.46*
	Rs2287780: C>T	0.0833	0.035	0.068	0.076
	Rs10380: C>T	0.1204	0.090	0.397	0.174
MAT1A	Rs114494303: G>A	0.01	0.00	NS	0.0002
	Rs72558181: G>A	0.01	0.00	NS	0.000009
	Rs112848063: A>G	0.00	0.00	NS	NA
	Rs116659053: G>A	0.00	0.00	NS	0.0002
MTR	Rs1805087: A>G	0.2091	0.220	0.981	0.218
CBS	Rs17849313: G>C	0.000	0.00	NS	NA
	Rs117687681: C>T	0.010	0.00	NS	NA
	Rs11700812: G>A/C	0.000	0.00	NS	NA

Global allele frequencies were derived from dbSNP147. *p* values were derived from chi-squared analysis with one degree of freedom

*p* values of significance (< 0.05) are in italics

*MAF* minor allele frequency, *NS* not significant, *NA* data not available

*The minor allele frequency was calculated from the mean MAF in EXAc (0.452) and Clinvar (0.47)

### Rare variants of *DNMT1* are present in some BWS cases with KCNQ1OT1 TSS-DMR LOM

**Fig. 2:**
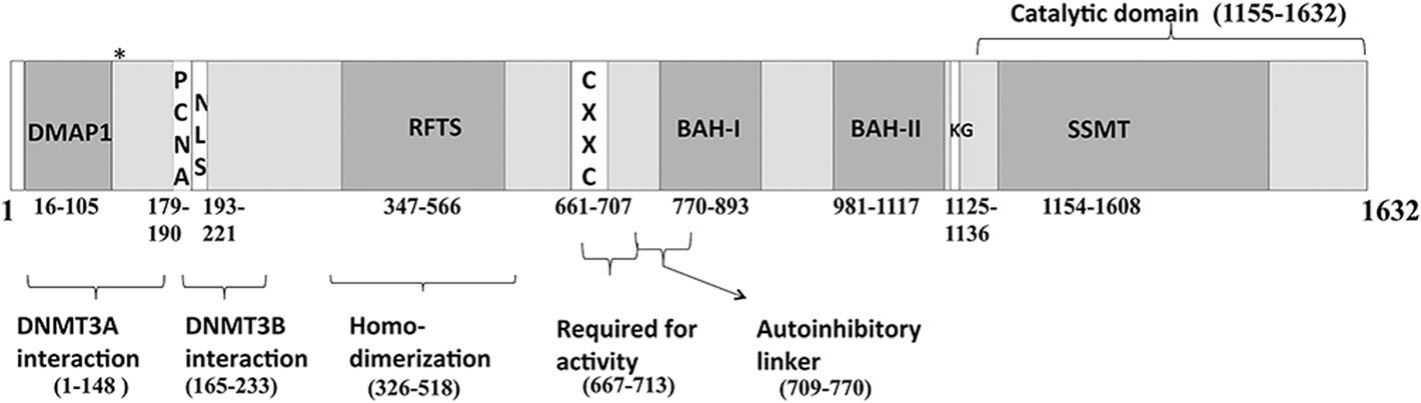
Structure of DNMT1 compiled from information in NCBI for NP_001124295.1 [45]. Replication fork targeting sequence (RFTS), nuclear localization signal (NLS), bromo-adjacent homology (BAH) domain, site-specific DNA cytosine methylase activity (SSMT), zinc finger domain (CXXC). The “*” symbol represents the translation start of the shorter DNMT1o form at amino acid 119. Conserved domains are DMAP1, RFTS, CXXC, BAH, KG linker sequence and SSMT. Numbers represent amino acid numbering

Dnmt1 and Dnmt1o proteins play a central role in the maintenance of DNA methylation during pre-implantation embryonic development, and DNMT1 is the major enzyme in the folate pathway responsible for the catalysis of *S*-adenosyl methionine, providing methyl groups for methylation of DNA. Hayward et al. [32] defined the location of human *DNMT1o* from sequence derived from EST BE537788. Primers were designed to this region (hg19 GRCh37 chr19: 10311482-10311816) and to the entire coding region of *DNMT1* (NM_001130823.1, representing the longest transcript). Variants affecting the coding regions of *DNMT1o* and *DNMT1* were screened by HRM and sequenced to confirm their identity. A schematic of DNMT1 and DNMT1o protein structure is shown in Fig. 2.

Fifty-three of the 55 BWS cases with KCNQ1OT1 TSS-DMR LOM were tested for SNVs in the coding regions of DNMT1. A total of 17 *DNMT1* SNVs were identified. No SNVs were identified in the sequence exon 1o, unique to *DNMT1* and upstream of the *DNMT1o* ATG. All of the *DNMT1* SNVs identified have been reported in dbSNP147. This database represents compiled variant data from 37 different genomic databases. Five substitutions were synonymous coding SNVs, six were located within the intronic sequence flanking exons and not predicted to affect splicing and one rare variant rs772176328: C>T, flanking exon 34 (NC_000019.10:g.10139820G>A (NM_001318731.1: c.3444-3C>T) had the potential to affect splicing but was not located within a canonical splice acceptor site. Five substitutions were missense variants with codon changes, four of which affected single patients only. Filtering of the missense *DNMT1* variants identified three with allele frequencies of less than 1 in 1000 making them potentially interesting candidates for a disorder with a population frequency of approximately 1 in 10,000 [33]. These were DNMT1 SNVs rs138841970: C>T, rs150331990: A>G and rs757460628: G>A encoding NP_001124295 p.Arg136Cys, p.His1118Arg and p.Arg1223His, respectively. Sequence traces are shown in Additional file 2: Figure S1. All missense variants identified and their allele frequencies are described in Table 3. All were heterozygous.

**Table 3:** Missense DNMT1 variants identified in BWS cases with LOM at KCNQ1OT1 TSS-DMR

SNV ID	Base change NM_001130823.1	AA change NP_001124295	Location NM_001130823.1	Ch37/Hg19 location	BWS VAF	dbSNP147 VAF	VEP
Rs 61750053	c.206G>A	p.Arg69His	Exon 3/41	Chr19:10291473	0.009	0.0089	Moderate
Rs 2228612	c.979A>G	p.Ile327Val	Exon 13/41	Chr19:10273372	0.11	0.135	Moderate
Rs 138841970	c.406C>T	p.Arg136Cys	Exon 4/41	Chr19:10291065	0.009	0.00028	Moderate
Rs 150331990	c.3353A>G	p.His1118Arg	Exon 31/41	Chr19:10251822	0.009	0.00001	Moderate
Rs 757460628	c.3668G>A	p.Arg1223His	Exon 33/41	Chr19:10250860	0.009	0.00002	Moderate

Variant allele frequencies were derived from dbSNP147. The variant effect predictor (VEP) tool was used to ascertain effects on protein function.

*VAF* variant allele frequency

All five missense variants identified, including the three rare missense variants, are predicted to have a moderate effect on protein function according to integrated analysis in Variant Effect Predictor (http://asia.ensembl.org/Tools/VEP). The variant, rs772176328: C>T, associated with a splice acceptor site, but not directly affecting a canonical base, was predicted to have a low impact on DNMT1 function. Amino acids Arg69 and Ile327 are not conserved in mouse, dog and some primates, and variants affecting these amino acid positions occur in control populations at frequencies of greater than 1 in 112 and 1 in 10, respectively, hence ruling them out as being significant in BWS. In contrast, Arg136 is conserved in all species expect dog, His1118 is conserved in all species except lamprey and Arg1223 is conserved in all species except mouse where it is leucine.

### Rare variants of *DNMT1* have reduced methyltransferase activity

**Fig. 3:**
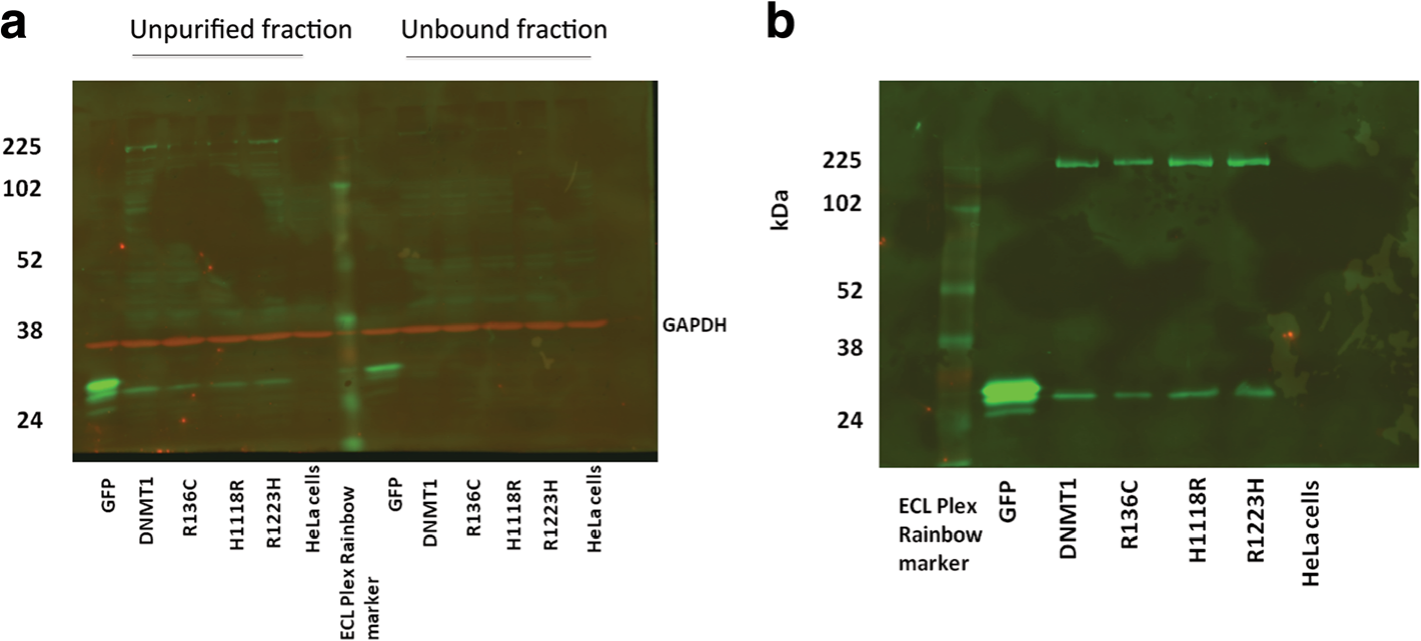
CyDye fluorescent Western blots. **a** HeLa cell protein lysates present in unpurified and unbound lysate fractions probed with primary antibodies anti-GFP and anti-GAPDH and with secondary antibodies goat-anti-rabbit IgG Cy5 and goat-anti-mouse IgG Cy3. **b** Purified GFP-tagged DNMT1 protein (DNMT1 225 kDa) and GFP (27 KDa) and DNMT1 variants generated by site-directed mutagenesis. The GFP bands in the fusion protein lanes in (**b**) likely represent cleavage products of the purified fusion protein

To directly examine the functional impact of the three rare DNMT1 missense variants, GFP-tagged DNMT1 recombinant proteins were generated and purified from Hela cells (Fig. 3 a, b).

**Fig. 4:**
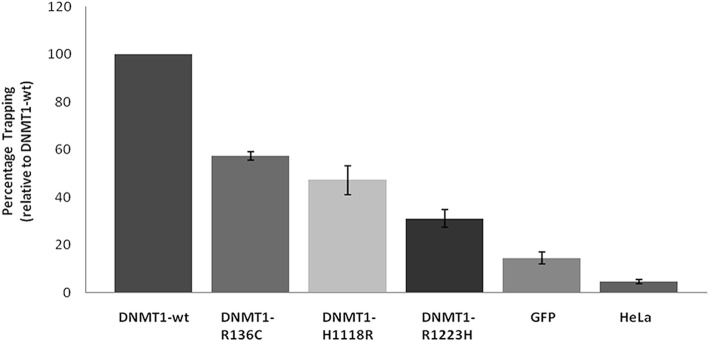
DNMT1 methyltransferase trapping activity. The relative trapping ability of each DNMT1 variant is shown as a percentage relative to wild type DNMT1. Proteins from pEGFP-C1 transfected HeLa cells and non-transfected HeLa cells acted as controls. Technical replicates were performed in each assay. Four biological replicate experiments were performed for each sample and controls, except for DNMT1-R136C variant for which only three biological replicate experiments were performed. Error bars: mean ± SE

Single nucleotide substitutions within *DNMT1* were inserted using site-directed mutagenesis (Additional file 2: Figure S2). Wild-type and DNMT1 mutant fusion proteins were expressed exclusively in the nucleus of Hela cells (Additional file 2: Figure S3). The functional impact of the rare missense variants on DNMT1 enzymatic activity was examined in a trapping assay previously described by Frauer and Leonhardt (2009) in which substrate is irreversibly covalently bound to DNMT1. The degree of binding is a surrogate for DNMT1 enzymatic activity as it measures the irreversible covalent enzyme-DNA complex formation as the first step of the DNA methylation reaction. Trapping activity measured relative to that of the wild-type DNMT1 protein is shown in Fig. 4.

The data in Fig. 4 shows that each missense variant exhibited reduced substrate trapping activity when compared with wild-type DNMT1 suggesting that these variants are less efficient at maintaining DNA methylation. Interestingly, each BWS case with these variants exhibited a complete LOM at KCNQ1OT1 TSS-DMR as opposed to partial or mosaic LOM the latter being a common finding in the majority of cases. The variants are predicted to affect the function of the N terminally truncated DNMT1 form, DNMT1o, in addition to DNMT1, although functional effects on DNMT1o were not examined directly. Information on these DNMT1 missense variants has been submitted to Clinvar and assigned accession numbers 3026604, 3027874 and 3028253.

It is interesting to note that *DNMT1* NM_001130823.1: c.406C>T, NP_001124295p.Arg136Cys, is located in the N-terminal domain and shows the least reduction in trapping activity whereas *DNMT1* NM_001130823.1: c.3668G>A, NP_001124295 p.Arg1223His, is located in the C-terminal catalytic domain very close to the catalytic site at p.Cys1242 and shows the maximum reduction in trapping activity compared to wild-type DNMT1 protein. The variant, *DNMT1* NM_001130823.1: c.3353 A>G, NP_001124295 p.His1118Arg, is located adjacent to the glycine-lysine repeat region linking the BAH2 domain to the C-terminal catalytic domain and has trapping ability of approximately 50% compared to the wild-type protein.

### Patient characteristics associated with variant *DNMT1*

Clinical features of patients with functionally impaired *DNMT1* variants of very low population frequency are shown in Table 4. BWS has a broad clinical presentation, and no particular feature could be ascribed to this subgroup. All cases had methylation values at KCNQ1OT1 TSS-DMR of 0.00 indicative of a complete loss of methylation in the blood. This is consistent with a defect affecting methylation establishment or maintenance very early in development either in the maternal germ cells or pre-implantation embryo. Only one of the three cases had loss of methylation at other imprinted loci consistent with MLID. Patient B96 had complete LOM at the *MEST* locus on chromosome 7q32.2 but maintained a normal methylation at *PLAGL1* (6q24), *GRB10* (7p12.1) *NESPAS* (20q13)) and *PEG3* (19q13). Interestingly, B96 had a brother who was stillborn, with an exomphalos and suspected BWS; however, this baby was born prior to the availability of molecular testing for BWS. Hence, for B96, there is a strong likelihood of an inherited predisposition. The mother of B97 was found to carry the R136C variant; however, she did not have features of BWS. Parental specimens from B96 and B66 were not available for testing. These patients also had less common variants in other folate pathway genes in varying combinations. All cases had at least two variant alleles corresponding to deleterious folate pathway polymorphisms in association with low-activity variants of DNMT1. These variants have been previously been shown to be associated with hypomethylation either dependent or independent of folate deficiency.

**Table 4:** Clinical features of BWS KCNQ1OT1 TSS-DMR LOM cases with rare DNMT1 missense variants.

Clinical feature	B66 (p.Arg1223His)	B96 (p.His1118Arg)	B97 (p.Arg136Cys)
Birth weight (kg)	3.1	4.54	3.43
Neonatal hypoglycaemia	Y	N	N
Exomphalos/umbilical hernia	Y	Y	N
Macroglossia	Y	Y	Y
Macrosomia	N	Y	Y
Ear creases	Y	N	Y
Malignancy	N	N	N
Facial naevus flammeus	N	N	N
Body asymmetry	N	N	N
Genitourinary abnormality	N	N	
Hepatomegaly	Y	N	N
Nephromegaly	N	N	N
ART	Y	N	N
Family history	N	Y (sibling affected)	N
Sex	F	F	M

## Discussion

In this paper, we have made observations that have not been previously reported. Firstly, patients with BWS and KCNQ1OT1 TSS-DMR LOM are more likely to have the low-activity *MTHFR* variant commonly known as c. 677C>T (rs1801133: C>T) although when applying a stringent statistical test for multiple testing, this association was not found to be statistically significant. BWS is a comparatively rare disorder, and examination of this relationship in larger cohorts will be required to consolidate it definitively. Secondly, we identified three rare missense variants of *DNMT1* and *DNMT1o* and characterised their capacity for maintaining DNA methylation in vitro. All variants had impaired enzymatic activity compared to wild-type *DNMT1*.

A large body of evidence has previously implicated *MTHFR* rs1801133: C>T in hyper-homocysteinaemia and DNA hypomethylation, that is potentiated by low dietary folate and homozygosity for the T allele [17–20, 22, 34–37]. *MTHFR* rs1801133: C>T causes an amino acid substitution of highly conserved alanine to valine at position 222 (p.Ala222Val) resulting in reduced enzyme activity due to increased thermolability [16]. Furthermore, BWS has been reported previously in association with homocysteinuria occurring with *CBS* enzyme deficiency [38]. Although we identified an association between the MTHFR rs1801133 T allele prevalence and BWS that was not statistically significant by a stringent statistical test, it remains to be determined whether a combination of low dietary folate and vitamin B6 and B12 deficiency, and this genotype may have a stronger association with BWS incidence. Future studies, including the prospective collection of dietary intake information, will require global collaborative efforts to examine these associations in large cohorts.

Our observations suggest that the identified low-activity *DNMT1* variants may contribute to a subgroup of BWS cases with imprinting defects through the effects on the maintenance methylation of hemimethylated DNA during DNA replication prior to implantation. Given the observation of complete loss of methylation in all three cases with variant *DNMT1*, we further propose that methylation maintenance failure at KCNQ1OT1 TSS-DMR occurs at a very early stage of embryogenesis in the cleavage embryo. Evidence from mouse models suggests a maternal effect on the maintenance of DNA methylation in the pre-implantation embryo. High levels of oocyte-derived maternal Dnmt1o are present in the cleavage embryo and protect imprinted loci from methylation loss at imprinted sites as well as in the intergenic regions. Mothers lacking Dnmt1o have abnormal imprinting in their offspring at the pre-implantation stage, including abnormal X-inactivation, in addition to placental hypomethylation [39]. Human *DNMT1o* is similarly expressed in mature oocytes and in early-stage embryonic development [32].

A direct role for DNMT1 in imprinting disorders as a result of maternal effect mutation is also suggested by the associations between the Uhrf1 protein and Dnmt1. Dnmt1 is recruited by Uhrf1 to replicating DNA [40], and intriguingly, a potentially deleterious UHRF mutation has been recently described as having a maternal effect in twins affected by MLID with loss of methylation [9]. Furthermore, another maternal effect protein, NLRP2, implicated in MLID and BWS, causes re-localization of Dnmt1 in oocytes and pre-implantation embryos [41]. Hence, there is accumulating evidence for the potential involvement of DNMT1 as a maternal effect protein associated with loss of methylation at imprinted loci.

Of the three cases with rare missense DNMT1 alleles, maternal inheritance of DNMT1 NM_001130823.1: c.406C>T, NP_001124295 p.Arg136Cys, could be demonstrated. Modes of inheritance could not be examined in the remaining two cases due to the lack of availability of parental DNA; however, case B96 had a sibling who was stillborn with exomphalos who was not tested for BWS, and the possibility of an inherited predisposition in this individual cannot be discounted. Case B66 with the variant DNMT1 NM_001130823.1: c.3668G>A, NP_001124295 p.Arg1223His, was a female conceived by intracytoplasmic sperm injection, and she also had a greater number of the clinical features of BWS when compared with the other two cases (five versus three in the other *DNMT1* variant cases). It is not known whether she had inherited the *DNMT1* variant from either of her parents; however, maternal inheritance would be consistent with recent observations in the mouse showing that oocytes deficient in *Dnmt1o* are more susceptible to imprinting defects following ART [42].

There was no reported indication of parental or familial BWS in any case. However, this might not be unexpected in a scenario where a DNMT1 mutation has a maternal effect in the pre-implantation embryo. Mothers may be mutation carriers themselves but unaffected if they had inherited a deleterious DNMT1 SNV from their fathers or had acquired one de novo. In such circumstances, only offspring arising from an oocyte expressing abnormal DNMT1 protein would be affected.

Previous studies on mouse *Dnmt1s* have identified regions required for maintenance of imprinting that are localised to the region spanning amino acids 191-394; however, none of the variants in this study are located within this domain [27]. The variant *DNMT1* NM_001130823.1: c.406C>T, NP_001124295 p.Arg136Cys, lies within an interaction site with DNMT3A suggesting the possibility of an effect on imprint establishment, given the evidence for coordination between DNMT1, DNMT3a and DNMT3b in both establishing and maintaining methylation [43, 44]. Recent studies have revealed insight into the structure of human DNMT1 protein, and conserved domains are described for DNMT1 NP_001124295.1 in the Conserved Domain Database hosted by NCBI [45–47]. None of the identified variants was located directly within the enzymatic active site at Cysteine 1242 in Ref Seq NP_001124295. Variant *DNMT1*: NP_001124295 p.His1118Arg is immediately adjacent to the second bromo-adjacent homology (BAH2) domain, and variant DNMT1: NP_001124295 p.Arg1223His lies within the catalytic domain. Histidine at 1118 is distant (> 45 Å away) from the DNA binding, SAM binding and catalytic cysteine within the catalytic domain. In the DNMT1 structures, this residue is in a short helix where it is solvent exposed and has few intra-molecular interactions (structure 3PTA in [47] and structure 4WXX in [46]). Residue Arg1223 is in the catalytic domain and distant from the DNA, SAM and Cys1242 sites (21 Å, < 15 Å, < 28 Å, respectively). This residue is leucine in mouse Dnmt1 (structure PDB ID 4DA4 [48], and both residues occupy similar structural space at the apex of a loop. In the human DNMT1 structures (PDB IDS 4WXX, 3PTA), the arginine adopts two radically side chain conformations. In both configurations, the arginine side chain does not engage in intra-molecular polar contacts and the residue is solvent exposed. The loop in which the DNMT1: NP_001124295 p.Arg1223His variant is found is added during the evolution of cytosine C5 methyltransferases. The loop is absent in the methyltransferase from Haemophilus parahaemolyticus (HhaI) [49] that has high structural similarity to the mouse and human DNMT1 catalytic domain, suggesting it is an acquired adaptation and not evolutionarily required for activity. Previous work found that *DNMT1* mutations that cause hereditary sensory neuropathy were due to protein misfolding [50]. One residue was in a hydrophobic core region while the other was in a linker region between secondary structural elements. Both mutations were found to affect the folding and stability of DNMT1 protein. In contrast, the variants reported here are in surface-exposed regions and are unlikely to be involved in the intra-molecular interactions. Therefore, they are not proposed to affect folding or stability. However, despite sharing 80% sequence identity, recent subtle differences in function between the mouse and human structures of DNMT1 protein have been recently recognised [46]. Therefore, any definitive structural effects of the identified variants are currently unclear. It may be possible that mutations exert their effects by altering conformational flexibility that is not apparent in the crystal structure. It is interesting to note however that the effect of the *DNMT1*: NP_001124295 p.Arg1223His variant on the activity was the strongest in the trapping assay and comparable to the effects described for *DNMT1* variants reported in the previous study examining hereditary neuropathies [50].

In a setting of maternal dietary folate deficiency or impaired embryonic folate metabolism, the observed decrease in DNMT1 enzymatic activity could be further exaggerated by reduced availability of the substrate *S*-adenosyl methionine (SAM) or increases in inhibitory *S*-adenosylhomocyteine (SAH). Interestingly, all cases with variant *DNMT1* had at least two variants predicted to adversely affect folate metabolism via the effects directly on *MTHFR* and methylation of tetrahydrofolate or via the effects on the conversion of homocysteine to methionine via methionine synthase reductase (MTRR) and 5-methyltetrahydrofolate-homocysteine *S*-methyltransferase (MTR) using vitamin B12 as a cofactor.

Due to their rarity in the general population, presence in a subgroup of BWS and MLID cases, and functional characterization supporting reduced methyltransferase activity, these DNMT1 variants are worthy of reporting. Ultimately, mouse models examining the effect of variant DNMT1 in the context of imprinting disruption at specific loci including KCNQ1OT1 TSS-DMR will be required for proof of association; however, larger cohort studies on families with imprinting disorders are also likely to be fruitful.

## Conclusions

In conclusion, the observations from this study suggest that novel genetic factors affecting DNA methylation may cause imprinting disruption in a subgroup of BWS cases. This study paves the way for larger prospective studies in BWS and other human imprinting disorders to examine the relationship between genotypes linked to DNA methylation in concert with environmental factors, to fully elucidate the causes of abnormal genomic imprinting.

## Methods

### Participant recruitment

The laboratory in which this work was conducted is the primary referral laboratory for the diagnosis of BWS in Australasia. After diagnostic testing for BWS using methylation-sensitive MLPA (MS-MLPA) to interrogate imprinting defects in 11p15.5, patients with evidence of sporadic loss of methylation at KCNQ1OT1 TSS-DMR were invited to participate in this research project. Parents were asked to complete a clinical questionnaire from which data on clinical features and fertility history were collected. The project and consent forms were approved by the Human Research Ethics Committee of the Royal Children’s Hospital in Melbourne (HREC 21121M), where the work was conducted. The participant recruitment extended over a period of 5 years.

Cord blood samples used as controls were obtained from the BMDI Cord Blood Bank at the Royal Children’s Hospital, Melbourne, Australia. These samples were deemed unsuitable for transplantation and approved for use in this project by the local institutional ethics committee.

### Molecular diagnosis of Beckwith-Wiedemann syndrome

Patients referred to the laboratory were assessed as having BWS with isolated loss of methylation at 11p15.5 KCNQ1OT1 TSS-DMR using the Salsa ME030-B1 BWS/RSS MS MLPA kit (MRC Holland). Patients with either partial or complete loss of methylation at KCNQ1OT1 TSS-DMR were included in the study. H19 methylation abnormalities and patUPD11p15 were not present in any cases included. DNA was extracted from EDTA blood using the Puregene kit (Qiagen Hilden Germany) and stored at − 20 °C prior to use.

### PCR and high-resolution melting

PCR was performed in a 20-μl total volume on all samples using HotStar Taq DNA Polymerase (Qiagen). Typical cycling conditions were 95 °C for 15 min followed by 40 cycles of 95 °C for 30 s, annealing temperature for 30s, extension at 72 °C for 45 s followed by a final extension at 72 °C for 5 min. HRM reactions were performed in a total volume of 20 μL and comprised 2× HRM Sensimix (Bioline) (10 μL), 0.50 μL of forward and reverse primers at 20 μM, 1.0 μL of DNA at 10 ng/μL. Amplification reactions were 95 °C for 10 min, followed by 40 cycles of 95 °C for 15 s, annealing temperature for 10 s, 72 °C for 10 s followed by 72 °C for 5 min. Primers used in PCR, HRM and sequencing are described in the Additional file 1: Tables S2a–e, S3a–e and S4a–c.

### Sanger sequencing

PCR products were treated with ExoSAP-IT (USB, Affymetrix) and sequenced with Big Dye Terminator v3.1 (BDT) mix (Applied Biosystems) according to the manufacturer’s instructions. Sequences were analysed with Mutation Surveyor v 4.0 (SoftGenetics, USA).

### Variant classification

Variants were examined using Polyphen (genetics.bwh.harvard.edu/pph2/), SIFT (sift.bii.a-star.edu.sg) Variant Effect Prediction tool (https://www.ensembl.org/vep) [51], Mutation Taster (www.mutationtaster.org/) and the University of Maryland Genetic Variant Interpretation tool.

SNVs are reported relative to their cDNA reference sequences where the nucleotide numbering uses + 1 as the A of the ATG translation initiation codon. Reference sequences used are as follows: *MTHFR* NM_005957.4, *MTRR* NM_002454.2, *MAT1A* NM_00429.2, *MTR* NM_000254.2, *CBS* NM_001178009.1 and *DNMT1* NM_001130823.1. The DNMT1 genomic reference sequence used for intronic variants was NC_000019.10.

### Analysis of methylation at other centres of genomic imprinting

The *PLAGL* (6q24), *GRB10* (7p12.1), *NESPAS* (20q13) and *PEG3* (19q13) loci were evaluated for methylation by pyrosequencing. Methylation at the MEST locus was evaluated by MS-MLPA.

Genomic DNA samples from 55 BWS cases with KCNQ1OT1 TSS-DMR LOM and from 13 controls were bisulphite modified using the MethylEasy DNA bisulphite modification kit (Cat No: ME-001, Human Genetic Signatures). Pyrosequencing PCR and sequencing primers were based on those described in Mackay et al. [52]. Primers for PCR are listed in Additional file 1: Table S5a, and primers used for sequencing are listed in Additional file 1: Table S5b. PCR was performed in a 40-μL total volume of 1× HotStar Master mix with 0.2 μL HotStar Taq DNA Polymerase (Cat. No: 203203, Qiagen), 0.2 μL of 20 μM forward and reverse primers, 5× Q solution (Qiagen) and 0.2 mM dNTPs. PCR cycling was at 95 °C for 15 min, followed by 45 cycles at 95 °C for 30 s, annealing temperature for 30 s and extension for 45 s at 72 °C. Pyrosequencing was performed on a PyroMark Q24 (Cat. #9001514, Qiagen) using PyroMark Q24 Gold (Cat. #970802, Qiagen) according to the manufacturer’s protocol. The results were analysed using PyroMark Q24 software to determine the methylation status. Thirteen control samples were used to establish the mean methylation values across the CpG sites examined. A normal range was considered to be the mean value ± two standard deviations from the mean. Patients were scored as abnormal if methylation was outside the established normal range. The *MEST* locus at 7q32.1 was examined using the SALSA MLPA kit ME032-A1 UPD7/UPD14 (MRC Holland) according to the manufacturer’s instructions. Two specimens previously reported with genome-wide mosaic uniparental isodisomy [53] were used as positive controls for loss of methylation. Loss of methylation at *MEST* was scored as positive when methylation was < 0.35.

### Generation of DNMT1 plasmids

The mammalian expression vector containing the long isoform of wild-type human DNMT1 with N-terminal GFP tag in pEGFP-C1 plasmid (Clontech) was obtained from Prof. Heinrich Leonhardt (Ludwig-Maximilians-University Biocentre, Munich). The pEGFP-C1-DNMT1 construct was transfected into competent DH5-alpha cells, and construct fidelity was assessed by sequencing plasmid DNA using primers designed to *DNMT1* exons as described in the supplementary information (cDNMT1 sequencing primers).

Bacterial cultures containing pEGFP-C1-DNMT1 were grown in 2YT and kanamycin and plasmid DNA extracted with either QIAprep Spin MiniPrep kit (Cat. # 27104, Qiagen) or Plasmid Maxi kit (Cat. #12162, Qiagen) appropriate to the scale of the culture and according to the manufacturer’s instructions.

### Site-directed mutagenesis

*DNMT1* coding variations were inserted into the wild-type *DNMT1* sequence in pEGFP-C1. Mutagenesis was performed with QuikChange II Site-Directed Mutagenesis kit (Cat. #200523, Agilent Technologies) according to the manufacturer’s instructions. Mutagenic primer pairs were designed for each identified *DNMT1* sequence variant using a web-based primer design tool (www.agilent.com/genomics/qcpd) provided by Agilent Technologies. The primers are listed following. Mutated sites are underlined:

*DNMT1*406C>T, F: CCAAACCCCTTTCCAAACCTTGCACGCCCAGG, R: CCTGGGCGTGCAAGGTTTGGAAAGGGGTTTGG; *DNMT1* 3353A>G, F: GATCCTCCCAACCGTGCCCGTA GCCCT, R: AGGGCTACGGGCACGGTTGGGAGGATC; and *DNMT1* 3668G>A F: CACCA ACTCCCACGGCCAGCGGC, R: GCCGCTGGCCGTGGGAGTTGGTG.

Following selection in kanamycin (50 μg/mL), white colonies derived from pEGFP-C1-DNMT1 mutants were selected and sequenced to confirm the presence of the mutation. Sequence traces from mutants are shown in Additional file 2: Figure S2.

### Transfection of DNMT1 into Hela cells

Wild-type *DNMT1* and *DNMT1* mutant plasmids were transfected into HeLa cells using FugeneHD (Promega) at a volumetric ratio of 3:1. Transfection efficiency was analysed by flow cytometry (BD LSR II, BD Biosciences) after 48 h. HeLa cells were transfected at a density of 1 × 10^5^ cells per well in a 12-well plate in 1 ml of DMEM containing 10% FCS (SAFC BioSciences) and incubated at 37 °C in 5% CO_2_. Nuclear GFP expression was visualised by confocal microscopy on a Leica TCS SP2. Transfection efficiencies of 60–70% were achieved.

### Purification of GFP-tagged DNMT1 proteins

The GFP-Trap_A kit (Chromotek) was used to purify GFP-tagged DNMT1 proteins from HeLa cells according to the manufacturer’s instructions.

### Protein analysis and Western blotting

Total protein estimation using the bichoninic acid (BCA) assay (Sigma-Aldrich) was performed on 20 μL cell lysate according to the manufacturer’s instructions. Western blotting with fluorescence visualization was performed as described in [54].

Purified GFP-tagged DNMT1 protein bound to beads was quantified using a DNMT1 ELISA kit (Epigentek Cat #P-3011). Standards were prepared from 7.5, 10, 12.5 and 15 ng of purified DNMT1 (supplied in the kit), and absorbance was read at 450 and 620 nm on a Multiskan EX microplate reader (Thermo Electron).

### Measurement of DNA methyltransferase activity

The DNA methyltransferase activity of the generated wild-type and mutant DNMT1 proteins was measured by an irreversible covalent interaction with a hemimethylated trapping substrate containing 5-aza-2′-deoxycytidine (5-azadC) as described in [55] and outlined below and in Additional file 2: Figure S4. This reaction is designed to irreversibly “trap” active DNMT1 in an inactive conformation by binding to the mechanism-based inhibitor 5-azadC. Capture and detection of this reaction intermediate serve as a measure of DNMT1 enzyme activity. Oligonucleotides used for the preparation of the DNA trapping substrate were synthesised by Integrated DNA Technologies. The forward oligo, MG-Upper contains a methylated cytosine (M) and the reverse 3′ end-paired Fill-in-oligo is labelled with a MAX-NHS ester fluorescent dye at its 5′ end. The Fill-in-oligo is complementary to the 3′ end of the MG-Upper oligonucleotide. Oligo sequences were as follows: MGUpper 5′CTCAACAACTAACTACCATCMGGACCAGAAGAGTCATCATGG 3′ and Fill-in 5′MAXN-CCATGATGACTCTTCTGGTC 3′.

Double-stranded DNA generated by oligo extension was prepared by denaturing 1 μL of each oligo at 100 μM in NEB2 buffer at 95 °C for 2 min followed by a slow cooling to 37 °C in a total final reaction volume of 15.2 μL. Hemimethylated DNA substrate was synthesised by extension of the fill-in-oligo with the addition of 1 μL DNA Polymerase I (Large Klenow) fragment (Cat #M2201, Promega), 1 mM each of dTTP, dATP and dGTP (Cat #BIO-39025, Bioline) and 50 μM 5-aza-dCTP (Cat #NU-1118S, Jena Bioscience) in acetylated BSA at 20 ng/μL (Cat #R3961, Promega) in a final reaction volume of 20 μL. The mix was incubated at room temperature for 10 min followed by inactivation at 75 °C for 10 min. This preparation of trapping substrate was used immediately in the in vitro DNMT1 trapping assay described below.

Fifteen nanograms of each purified pull-down GFP-tagged DNMT1 protein (bead-bound) derived from each DNMT1 protein variant was resuspended in buffer (100 mM KCl, 10 mM Tris-HCl, pH 7.6, 1 mM EDTA, 1 mM DTT) supplemented with 100 μM *S*-adenosyl-l-methionine (SAM) (New England Biolabs, Cat #B9003S), 4 μL end-labelled DNA trapping substrate (prepared as described above) and 160 ng/μL BSA in a total reaction volume of 200 μL. For determination of DNA methyltransferase activity, trapping was performed at 37 °C for 90 min with constant mixing. To remove unbound substrate, beads were washed twice with 1 mL of assay buffer, then resuspended in 100 μL of assay buffer and transferred to a 96-well microplate. Bound-labelled DNA trapping substrate associated with each variant and wild-type DNMT1 protein was measured by fluorescence emission on a FLUOstar Optima microplate reader (BMG Labtech). Technical duplicates were tested in each assay, and assays were performed in quadruplicate for each variant (except for DNMT1 Arg136Cys for which triplicate assays were performed) and the mean fluorescence for each DNMT1 variant was normalised to the wild-type DNMT1 fluorescence value, set to 100%. A schematic representation of the preparation of the DNA suicide substrate and the trapping assay is shown in Additional file 2: Figure S4.

### Statistical analysis

Calculations of chi-squared and significance values were calculated using online analysis tools including GraphPad. Allele association was calculated using a 2 × contingency table with one degree of freedom [56]. Probability values of *p* < 0.05 were considered to be statistically significant. Pearson values for chi-squared and probability were calculated. Bonferroni correction was also applied for the analysis of folate pathway SNVs to adjust for multiple testing.

## Additional files

Additional file 1: **Table S1.** Methylation at non-11p15 imprinting centres in BWS cases with loss of methylation at KCNQ1OT1 TSS-DMR. Only samples with identified methylation changes are listed. **Tables S2.** a–e Primers used for HRM of MTHFR, MTR, MTRR, MAT1A, CBS. **Table S3.** a–e Primers used for sequencing MTHFR, MTR, MTRR, MAT1A, CBS. **Table S4.** a–d. DNMT1 primers. **Table S5** Pyrosequencing primers. (DOCX 226 kb)

Additional file 2: **Figure S1.** DNMT1 sequence variants identified in BWS patients. **Figure S2.** Sequence traces of DNMT1 variants generated by site-directed mutagenesis. **Figure S3.** Expression of GFP-tagged DNMT1 proteins in HeLa cells. **Figure S4.** Schematic of the trapping assay adapted from Frauer and Leonhardt (2009). (PPTX 1525 kb)

## Abbreviations

BWS: Beckwith-Wiedemann syndrome
CBS: Cystathionine beta-synthase
DNMT1: DNA methyltransferase 1
*MAT1A*: Methionine adenosyltransferase
MLID: Multi-locus imprinting disruption
*MTHFR*: Methylenetetrahydrofolate reductase
MTR: 5-Methyltetrahydrofolate-homocysteine *S*-methyltransferase
MTRR: Methionine synthase reductase
SAH: *S*-adenosyl-homocysteine
SAM: *S*-adenosyl-methionine
SNV: Single nucleotide variant
