# Supplementary material for: Genetic variation affecting DNA methylation and the human imprinting disorder, Beckwith-Wiedemann syndrome
Source: Clin Epigenetics. 2018 Aug 30;10:114. doi: 10.1186/s13148-018-0546-4 (PMC6117921; doi:10.1186/s13148-018-0546-4)
Supplement: Supplementary file 1 — Table S1. Methylation at non-11p15 imprinting centres in BWS cases with loss of methylation at KCNQ1OT1 TSS-DMR. Only samples with identified methylation changes are listed. Tables S2. a–e Primers used for HRM of MTHFR, MTR, MTRR, MAT1A, CBS. Table S3. a–e Primers used for sequencing MTHFR, MTR, MTRR, MAT1A, CBS. Table S4. a–d. DNMT1 primers. Table S5 Pyrosequencing primers. (DOCX 226 kb) [file 13148_2018_546_MOESM1_ESM.docx]

Table S2a-e

**Primers used for HRM of MTHFR, MTR, MTRR, MAT1A, CBS.**

**a**

| **SNP** | **Primer sequence** | **Ta (⁰C)** | **Size (bp)** | **HRM range (⁰C)** | **GRCh37/hg19 coordinates** |
| --- | --- | --- | --- | --- | --- |
| *MTHFR* 677C>T (rs1801133) | F:TGAAGCACTTGAAGGAGAAGG | 55 | 59 | 67 - 82 | chr1:11856353-11856411 |
|  | R:GAAAAGCTGCGTGATGATGA |  |  |  |  |
| *MTHFR* 1298A>C (rs1801131) | F: GGGGAGGAGCTGACCAGT | 57 | 52 | 65 - 80 | chr1:11854447-11854498 |
|  | R:GGTAAAGAACCAAGACTTCAAAGA |  |  |  |  |
| *MTHFR* 1793G>A (rs2274976) | F: CTTTGCCCTGTGGATTGAG | 55 | 53 | 68 - 83 | chr1:11850895-11850947 |
|  | R: ACGGGGACTCCTCCTCATAC |  |  |  |  |

**b**

| **SNP** | **Primer sequence** | **Ta (⁰C)** | **Amp size (bp)** | **HRM**  **range (⁰C)** | **GRCh37/hg19**  **coordinates** |
| --- | --- | --- | --- | --- | --- |
| *MTR* 2756A>G (rs1805087) | F:TGGAAGAATATGAAGATATTAGACAGG | 55 | 59 | 62 - 77 | chr1:237048473-237048531 |
|  | R:TCTACCACTTACCTTGAGAGACTCAT |  |  |  |  |

**c**

| **SNP** | **Primer sequence** | **Ta (⁰C)** | **Amp size (bp)** | **HRM**  **range (⁰C)** | **GRCh37/hg19**  **coordinates** |
| --- | --- | --- | --- | --- | --- |
| *MTRR* 66A>G (rs1801394) | F: AAGGCCATCGCAGAAGAAAT | 55 | 55 | 65 - 80 | chr5:7870953-7871007 |
|  | R:CTGCAGAAAATCCATGTACCA |  |  |  |  |
| *MTRR* 25088C>T (rs2287780) | F:CGAGCCCTTGTGGACTATACC | 53 | 116 | 72-87 | chr5:7889217-7889332 |
|  | R: AAGCAGGCACAGGCATCT |  |  |  |  |
| *MTRR* 32975C>T (rs10380) | F:TGACAACCTTTTAGTGATCCATT | 55 | 75 | 60-75 | chr5:7897139-7897213 |
|  | R:AAGATCCCATGCTTAAGGAAA |  |  |  |  |

**d**

| **SNP** | **Primer sequence** | **Ta (⁰C)** | **Amp size (bp)** | **HRM range (⁰C)** | **GRCh37/hg19**  **coordinates** |
| --- | --- | --- | --- | --- | --- |
| *MAT1A* 531C>A (rs79744925) | F:TGAGGGACACCATCAAGCACAT | 63 | 52 | 67-83 | [chr10:82043665-82043716](http://genome.ucsc.edu/cgi-bin/hgTracks?hgsid=309504039&db=hg19&position=chr10:82043665-82043716&hgPcrResult=pack) |
|  | R:CCCTCACCCTTGGCTGAGTCAT |  |  |  |  |
| *MAT1A* 785G>A (rs114494303) | F: CCGGATGGCAGACCTCAG | 57 | 86 | 72-87 | [chr10:82039907-82039992](http://genome.ucsc.edu/cgi-bin/hgTracks?hgsid=309504039&db=hg19&position=chr10:82039907-82039992&hgPcrResult=pack) |
|  | R:CTGGGGTAATTCAGCTGCTC |  |  |  |  |
| *MAT1A* 1046G>A (rs72558181) | F:ATCTTGATTTCTCTCCCTCTGC | 61 | 73 | 65-85 | [chr10:82034907-82034979](http://genome.ucsc.edu/cgi-bin/hgTracks?hgsid=309504039&db=hg19&position=chr10:82034907-82034979&hgPcrResult=pack) |
|  | R:CGCCATAGGTGTCCACAATAAT |  |  |  |  |
| *MAT1A* 1316A>G (rs112848063)  & 1321C>T (rs116659053) | F:GCTGCTGGATGTGGTGCATA | 59 | 64 | 70-85 | [chr10:82034266-82034329](http://genome.ucsc.edu/cgi-bin/hgTracks?hgsid=309504039&db=hg19&position=chr10:82034266-82034329&hgPcrResult=pack) |
|  | R:GGCACTTTACCTGACAATGACG |  |  |  |  |

**e**

| **SNP** | **Primer sequence** | **Ta (⁰C)** | **Amp size (bp)** | **HRM range (⁰C)** | **GRCh37/hg19 coordinates** |
| --- | --- | --- | --- | --- | --- |
| *CBS* 450G>C (rs17849313) | F: CGAGTCCCCACATCACCAC | 59 | 71 | 67-85 | [chr21:44492051-44492121](http://genome.ucsc.edu/cgi-bin/hgTracks?hgsid=309504039&db=hg19&position=chr21:44492051-44492121&hgPcrResult=pack) |
|  | R:CCAGGTTATGGATCAGCCCTCT |  |  |  |  |
| *CBS* 1350C>T (rs117687681)  and 1351G>A/C (rs11700812) | F: GCGCAGGAGCTGCAGGAG | 59 | 54 | 73-88 | [chr21:44480562-44480615](http://genome.ucsc.edu/cgi-bin/hgTracks?hgsid=309504039&db=hg19&position=chr21:44480562-44480615&hgPcrResult=pack) |
|  | R:CACTGAGTCGGGCAGAATGAC |  |  |  |  |

Table S3 a-e.

**Primers used for sequencing** **MTHFR, MTR, MTRR, MAT1A, CBS**

| **SNP ID** | **Primer sequence** | **Ta (⁰C)** | **Size (bp)** | **GRCh37/hg19 coordinates** |
| --- | --- | --- | --- | --- |
| rs1801133 | F:GCCAGCCTCTCCTGACTGTCATCCCT | 65 | 250 | chr1:11856241-11856490 |
|  | R: TGGGCTCTCCTGGGCCCCTCA |  |  |  |
| rs1801131 | F: GCCCTGACCTCTGGGCACCCCT | 65 | 242 | chr1:11854383-11854624 |
|  | R:TGAACCAGGGTCCCCACTCCAGCA |  |  |  |
| rs2274976 | F:TGGGCAGGGGTGGGAAGTGATACTGGC | 65 | 213 | chr1:11850889-11851101 |
|  | R:TGCGGGACGGGGACTCCTCCTCAT |  |  |  |

**a. MTHFR**

**b. MTR**

| **SNP ID** | **Primer sequence** | **Ta (⁰C)** | **Size (bp)** | **GRCh37/hg19) coordinates** |
| --- | --- | --- | --- | --- |
| rs1805087 | F:TGCTCATCTATGGCTATCTTGC | 59 | 157 | chr1:237048391-237048547 |
|  | R:CAAGCAAAAATCTGTTTCTACCA |  |  |  |
|  |  |  |  |  |

**c. MTRR**

| **SNP ID** | **Primer sequence** | **Ta (⁰C)** | **Size (bp)** | **GRCh37/hg19) coordinates** |
| --- | --- | --- | --- | --- |
| rs1801394 | F:TACTGCTTCATTAAAAAGAGGATCT | 59 | 232 | chr5:7870839-7871070 |
|  | R:ACAAAACGGTAAAATCCACTGTAA |  |  |  |
| rs2287780 | F: CGAGCCCTTGTGGACTATACC | 53 | 116 | chr5:7889217-7889332 |
|  | R: AAGCAGGCACAGGCATCT |  |  |  |
| rs10380 | F:CAATTCCAGTATTGTACTCAACCAC | 55 | 136 | chr5:7897078-7897213 |
|  | R: AAGATCCCATGCTTAAGGAAA |  |  |  |

**d. MAT1A**

| **SNP ID** | **Primer sequence** | **Ta (⁰C)** | **Amp size (bp)** | **GRCh37/hg19) coordinates** |
| --- | --- | --- | --- | --- |
| rs79744925 | F: GGCACTTGGCTGCTAACTCT | 59 | 173 | [chr10:82043665-82043837](http://genome.ucsc.edu/cgi-bin/hgTracks?hgsid=309504039&db=hg19&position=chr10:82043665-82043837&hgPcrResult=pack) |
|  | R: CCCTCACCCTTGGCTGAGTCAT |  |  |  |
| rs114494303 | F: CATCCTTGCTCACAAGCTCA | 57 | 110 | [chr10:82039907-82040016](http://genome.ucsc.edu/cgi-bin/hgTracks?hgsid=309504039&db=hg19&position=chr10:82039907-82040016&hgPcrResult=pack) |
|  | R: CTGGGGTAATTCAGCTGCTC |  |  |  |
| rs72558181 | F: AGTACTTCAGCCCCCTCCAG | 61 | 118 | [chr10:82034907-82035024](http://genome.ucsc.edu/cgi-bin/hgTracks?hgsid=309504039&db=hg19&position=chr10:82034907-82035024&hgPcrResult=pack) |
|  | R: CGCCATAGGTGTCCACAATAAT |  |  |  |
| rs112848063  and rs116659053 | F: CAGAGGCTTCAATCCCTGAC | 59 | 182 | [chr10:82034266-82034447](http://genome.ucsc.edu/cgi-bin/hgTracks?hgsid=309504039&db=hg19&position=chr10:82034266-82034447&hgPcrResult=pack) |
|  | R: GGCACTTTACCTGACAATGACG |  |  |  |

**e. CBS**

| **SNP ID** | **Primer sequence** | **Ta (⁰C)** | **Amp size (bp)** | **GRCh37/hg19 coordinates** |
| --- | --- | --- | --- | --- |
| rs17849313 | F: GGTCCCCAGAGGATAAGGAA | 59 | 162 | [chr21:44492051-44492212](http://genome.ucsc.edu/cgi-bin/hgTracks?hgsid=309504039&db=hg19&position=chr21:44492051-44492212&hgPcrResult=pack) |
|  | R: CCAGGTTATGGATCAGCCCTCT |  |  |  |
| rs117687681 and rs11700812 | F: GTGCACAATTCATGCATACG | 55 | 169 | [chr21:44480562-44480730](http://genome.ucsc.edu/cgi-bin/hgTracks?hgsid=309504039&db=hg19&position=chr21:44480562-44480730&hgPcrResult=pack) |
|  | R: CACTGAGTCGGGCAGAATGAC |  |  |  |

Table S4a-d

DNMT1 primers

Primers used for HRM mutation scanning of the DNMT1 gene are shown in Table 3a below.

Not all DNMT1 exons were suitable for HRM scanning and several were sequenced directly.

The primer pair for Exon 4 was designed so that the amplicon includes the alternative Exon 5 that forms part of the longer isoform (a) of *DNMT1* (mRNA reference: NM_001130823).

**a. DNMT1 HRM primers**

| **Exon** | **Primer sequence** | **Ta (⁰C)** | **Amp size (bp)** | **HRM range (⁰C)** | **GRCh37/hg19) coordinates** |
| --- | --- | --- | --- | --- | --- |
| *DNMT1*  Exon 2 | F: CAGGCAAATGAAGTTGCTTAAC | 57 | 156 | 65 - 80 | >chr19:10292657-10292812 |
|  | R: TGAGCCACAAAGTGTGTCAG |  |  |  |  |
| *DNMT1*  Exon 3 | F: CCTGCCATTCACTCATTGTG | 57 | 245 | 67 - 82 | >chr19:10291428-10291672 |
|  | R: GGCTAGGATGCTGAGAACTGA |  |  |  |  |
| *DNMT1*  Exon 6 | F: GAACCTGGCTTCCTGCAATA | 55 | 213 | 70 - 85 | >chr19:10287942-10288154 |
|  | R: CAGCCGCCAATTTATCGTAT |  |  |  |  |
| *DNMT1*  Exon 7 | F: GGAGGCCTAGGGTCTTGTTC | 55 | 156 | 72 - 87 | >chr19:10286190-10286345 |
|  | R: TCAGGAAATGAAAGCACTGG |  |  |  |  |
| *DNMT1*  Exon 8 | F: GGTGTTGACCTTCCCTTGA | 55 | 184 | 67 - 82 | >chr19:10284461-10284644 |
|  | R: CCCGGCCTTAAACTTTCTG |  |  |  |  |
| *DNMT1*  Exon 9 | F:AGCTCCTGGCACTCACACTTGGGGT | 65 | 221 | 70 - 85 | >chr19:10283676-10283896 |
|  | R: CCCCACCCCCTGTCCCCACGTCC |  |  |  |  |
| *DNMT1*  Exon 10 | F: TTTACTCCCACCAGTGGACTC | 55 | 187 | 67 - 82 | >chr19:10278973-10279159 |
|  | R: AACAAAGCACAAAGGCAGGT |  |  |  |  |
| *DNMT1*  Exon 11 | F: GACCAGCCTGTGTGTGTGTT | 59 | 167 | 72 - 87 | >chr19:10277229-10277395 |
|  | R: GCAGAAGAATGAGGGGGAGT |  |  |  |  |
| *DNMT1*  Exon 12 | F: GCATTTTATCTCACTCTGGCTCA | 59 | 186 | 67 - 82 | >chr19:10273929-10274114 |
|  | R: CACCACACCTGGCCTAGAAC |  |  |  |  |
| *DNMT1*  Exon 14 | F: CCGAAGCACTATCTGGGTGT | 55 | 205 | 67 - 82 | >chr19:10270956-10271160 |
|  | R: CCTAAGGTTGCTCTGGCAAT |  |  |  |  |
| *DNMT1*  Exon 16 | F: GCCGGTGGCGGCGCTCACGA | 65 | 219 | 75 - 90 | >chr19:10270453-10270671 |
|  | R: ACGGGGCTGGTGAGCAGTGGG |  |  |  |  |
| *DNMT1*  Exon 17 | F: TCCCACTGCTCACCAGCCCCGT | 65 | 208 | 82 - 87 | >chr19:10270267-10270474 |
|  | R: GCCCGCAGGCACCTCTGGGGATG |  |  |  |  |
| *DNMT1*  Exon 18 | F: AGCATGTGCTTTGTTTCCTGT | 57 | 179 | 70 - 85 | >chr19:10267032-10267210 |
|  | R: GTGTGCCCCAAACATAATCC |  |  |  |  |
| *DNMT1*  Exon 19 | F: CTGGGTTTGGGATGTGAGTC | 59 | 170 | 70 - 85 | >chr19:10266506-10266675 |
|  | R: CCTATGATGGGCCACACACT |  |  |  |  |
| *DNMT1*  Exon 20 | F: CCCTTTTCCACACCTCCTCT | 57 | 245 | 72 - 87 | >chr19:10265519-10265763 |
|  | R: GCTGGTTCTGAAGGCAAGTT |  |  |  |  |
| *DNMT1*  Exon 21 | F: TGGCTTAAGCATGAGAGAAACC | 61 | 266 | 75 - 90 | >chr19:10265220-10265485 |
|  | R: CCCGGTCTCCAGTCTTCACT |  |  |  |  |
| *DNMT1*  Exon 22 | F: GTCCCAGTCCTCTGAGATGC | 59 | 245 | 75 - 90 | >chr19:10264948-10265192 |
|  | R: AGCACCCACAGGTGAGGTTA |  |  |  |  |
| *DNMT1*  Exon 23 | F: TCGGCCTTCTGAAATACTGG | 57 | 210 | 75 - 90 | >chr19:10262394-10262603 |
|  | R: CTGCCACAGGAGGAAGACTC |  |  |  |  |
| *DNMT1*  Exon 24 | F: GCCCCGGTTGGTCTTACTTA | 55 | 250 | 72 - 87 | >chr19:10262028-10262277 |
|  | R: TATTGGGAACATGGCAGTGA |  |  |  |  |
| *DNMT1*  Exon 25 | F: ACCCTCAGAATGATCCTCCA | 55 | 194 | 68 - 83 | >chr19:10260494-10260687 |
|  |  |  |  |  |  |
|  | R: GCATGCAGAAGTCAAGCAAA |  |  |  |  |
| *DNMT1*  Exon 27 | F: GGCCTGACACGCATTCTTAT | 57 | 217 | 75 - 90 | >chr19:10259505-10259721 |
|  | R: GGAGTGATGTGGGCTGAAAG |  |  |  |  |
| *DNMT1*  Exon 29 | F: TGATGTCAAGTGGGTGCTTC | 57 | 293 | 75 - 90 | >chr19:10254398-10254690 |
|  | R: AGAAGAGTCTGTGGCCTCGT |  |  |  |  |
| *DNMT1*  Exon 30 | F: CTCCTAACGAGGCCTCTCC | 57 | 250 | 77 - 92 | >chr19:10252675-10252924 |
|  | R: GCCTTCCCTCTAGCAAGCA |  |  |  |  |
| *DNMT1*  Exon 31 | F: CTGCGTTAGAGAGGGGACAG | 55 | 174 | 70 - 85 | >chr19:10251753-10251926 |
|  | R:TTGAAAAGAAACTCATACAATGACG |  |  |  |  |
| *DNMT1*  Exon 32 | F: GGTGGACTTGGACTCTGAGG | 59 | 226 | 75 - 90 | >chr19:10251412-10251637 |
|  | R: TCTGGGCTCACCAGGTATTC |  |  |  |  |
| *DNMT1*  Exon 36 | F: GGTGTTGACCTCCTCCTCTG | 59 | 225 | 75 - 90 | >chr19:10248487-10248711 |
|  | R: GCTCTGTCAGGGTGCCATTA |  |  |  |  |
| *DNMT1*  Exon 37 | F: TGCTCACATGAGGTGACAGC | 59 | 267 | 77 - 92 | >chr19:10247732-10247998 |
|  | R: CACCGGGAACCACAACTTAC |  |  |  |  |
| *DNMT1*  Exon 38 | F: TCATCACTGTCCTGTCTTCCA | 59 | 248 | 77 - 92 | >chr19:10246738-10246985 |
|  | R: AGTTAGGCCGCCAAGTACAG |  |  |  |  |
| *DNMT1*  Exon 39 | F: ATGCTGTCACAGTGCCATTT | 56 | 250 | 77 - 92 | >chr19:10246369-10246618 |
|  | R: AGCCTCCTTCCTGTCCAGA |  |  |  |  |
| *DNMT1*  Exon 40 | F: CATTGCCCTTCTCCCCTCCT | 64 | 218 | 73 - 88 | >chr19:10244837-10245007 |
|  | R: ATGTACCCCCAGAGGGCAGTC |  |  |  |  |

**b. DNMT1 PCR primers**

| **Exon** | **Primer sequence** | **Ta (⁰C)** | **Amp size (bp)** | **GRCh37/hg19) coordinates** |
| --- | --- | --- | --- | --- |
| *DNMT1*  Exon 1 | F: ACCCTTGGGGGCTTCTCGCTGCT | 65 | 482 | >chr19:10305447-10305928 |
|  | R: AGCGCCCTGCCTGTCCCCCT |  |  |  |
| *DNMT1*  Exon 4&5 | F: TGATGATGATGAAGGAAACACA | 58 | 491 | >chr19:10290844-10291334 |
|  | R: TGGGTGTGGTGGCACATA |  |  |  |
| *DNMT1*  Exon 13 | F: TGGGGACCCTGGGGCCTCTGA | 65 | 380 | >chr19:10273227-10273606 |
|  | R: TGCGCCACCACGCCCAGTCTTC |  |  |  |
| *DNMT1*  Exon 15 | F:GCCACAGGCACCTCAGGCAGAAACCT | 65 | 250 | >chr19:10270586-10270835 |
|  | R:TGGGAGGGTGGGTCTGTGGGAGCA |  |  |  |
| *DNMT1*  Exon 26 | F:CTAGCAAGGTTTGCATCTTTCTTT | 59 | 559 | >chr19:10259978-10260536 |
|  | R:GCAACAAGAGCGAAACTCAGTC |  |  |  |
| *DNMT1*  Exon 28 | F: AAAAGTTGCCTGTTTTTCTTGCT | 59 | 574 | >chr19:10256851-10257424 |
|  | R:CATCATGGTCAGTCTACACGATTT |  |  |  |
| *DNMT1*  Exon 33 | F: TGAGCCAAGATCACACCACTG | 61 | 606 | >chr19:10250515-10251120 |
|  | R: GCAGGAGTCACCTCCACAGACA |  |  |  |
| *DNMT1*  Exon 34 | F: GTGCCGCATGTCAGCAGTGGT | 65 | 525 | >chr19:10250152-10250676 |
|  | R: CACTAGCGGTGGACTTGCTGGG |  |  |  |
| *DNMT1*  Exon 35 | F:AAACTCCTCTGACCAGGAGCACG | 65 | 514 | >chr19:10248939-10249452 |
|  | R:GCAGGGTCAGAACTGGAGACCA |  |  |  |
| *DNMT1*  Exon 41 | F: GCCTCTGGGTCTAGAACCCTC | 59 | 633 | >chr19:10243950-10244582 |
|  | R: CAGCATGGAAGAGCTTGTTCC |  |  |  |

**c. DNMT1o PCR primers**

| **Exon** | **Primer sequence** | **Ta (⁰C)** | **Size (bp)** | **GRCh37/hg19) coordinates** |
| --- | --- | --- | --- | --- |
| *DNMT1o*  Exon 1o | F: TATTTCCTCCCCTGGCTCTAA | 59 | 335 | chr19:10311482-10311816 |
|  | R: TAGGTAGCAGGACCCTGTTTG |  |  |  |

**d. cDNMT1 sequencing primers for expression construct validation**

| **Exon** | **PCR Primer sequence (5' - 3')** | **Size (bp)** | **Amplicon location (NM_001130823.1)** |
| --- | --- | --- | --- |
| *pEGFP-C1 - DNMT1*  Exon 2 | F: CATGGTCCTGCTGGAGTTCGTG | 387 | 1266 (pEGFP-C1) - 266 (Ref mRNA) |
|  | R: ttgagccgcctgcggacatcgt |  |  |
| *DNMT1*  5’UTR -Exon 4 | F: TCGCCCCTCCCCATCGGTTT | 504 | 103 - 606 |
|  | R: CTTGCTCCTCCTGGGCGTGC |  |  |
| *DNMT1*  Exon 4 -Exon 11 | F: GGAACCAAGCAAGAAGTGAAGCCCGT | 520 | 508 - 1027 |
|  | R: TGGCTTCTCTGTCCGGCTCCTCCT |  |  |
| *DNMT1*  Exon 8 -Exon 17 | F: GACGTAGAGTTACATCCAGAGAACG | 591 | 839 - 1429 |
|  | R: CCTCATAACTCTCAAAGCCAGACT |  |  |
| *DNMT1*  Exon 16 -Exon 21 | F: ACCCACCAGACGCGGTGGAT | 564 | 1337 - 1900 |
|  | R: CCACAAACTGCGCGTGTCGC |  |  |
| *DNMT1*  Exon 21 -Exon 24 | F: ACCACGGTTCCTCCTTCTGGCCTCA | 617 | 1825 - 2441 |
|  | R: CGGCTTCTCCGACCCAAGAGATGCG |  |  |
| *DNMT1*  Exon 23 -Exon 28 | F: GCAGGCTTGCCAAGAGCGGA | 653 | 2277 - 2929 |
|  | R: CAGCCAGACGGGCACAGCTC |  |  |
| *DNMT1*  Exon 26 -Exon 30 | F: GCCCCCTCCGAAAACTGGGC | 665 | 2740 - 3404 |
|  | R: CGGCCCTGCACAGCCTTGAA |  |  |
| *DNMT1*  Exon 29 -Exon 33 | F: CCTGGATGCCCCTGAGCCCT | 601 | 3180 -3780 |
|  | R: GGAGCCGGGGTTGTTCAGCC |  |  |
| *DNMT1*  Exon 32 -Exon 35 | F: CTGTGAGCCGAGCGAGCCAG | 630 | 3600 - 4229 |
|  | R: AACACGTGCAGTGGCTCCGG |  |  |
| *DNMT1*  Exon 34 -Exon 37 | F: GAAGCTCACCCTCCGCTGCC | 520 | 4068 - 4587 |
|  | R: CCTGGCCATGGTGCCGTCTG |  |  |
| *DNMT1*  Exon 37 -Exon 41 | F: ATGCGGCACATCCCCTTGGC | 599 | 4501 - 5099 |
|  | R: GGGGTGACGGGAGGGCAGAA |  |  |
| *DNMT1*  Exon 40 –pEGFP-C1 | F: tgccgtgccaccgcccctgg | 540 | 4962 (Ref mRNA) - 1492 (pEGFP-C1) |
|  | R: GTTTCAGGTTCAGGGGGAGGTGTGGG |  |  |
| *DNMT1*  Exon 40 –pEGFP-C1  EBV-Rev primer | F: tgccgtgccaccgcccctgg | 676 | 4962 (Ref mRNA) - 1629 (pEGFP-C1) |
|  | R: GATGAGTTTGGACAAACCAC |  |  |
| pEGFP-C1 | F: CATGGTCCTGCTGGAGTTCGTG | 228 | pEGFP-C1 location: 1266-1493 |
|  | R: GTTTCAGGTTCAGGGGGAGGTGTGGG |  |  |

Table S5a and b

a. Primers used for amplification of products for pyrosequencing.

| **Imprinted Locus** | **Primer sequence (5' - 3')** | **Ta (⁰C)** | **Size (bp)** | **GRCh37/hg19 coordinates** |
| --- | --- | --- | --- | --- |
| *TND* | F: GAGGAGGGTGTGTTTTTGT | 53 | 177 | chr6:144328953-144329129 |
|  | R:AATCTATAAACCTCATACCAAATAAAC  (5’ biotinylated) |  |  |  |
| *GRB10* | F:GGTTTTGGAGTATAATAGGAATTT  (5’ biotinylated) | 55 | 115 | chr7:50850569+  50850683 |
|  | R:ATTACCATAAAAACCAAAAATCC |  |  |  |
| *NESPAS* | F: GATGAAGGGGTGGTTAGTA  (5’ biotinylated) | 53 | 229 | chr20:57430016+  57430244 |
|  | R:CCAAAAATACCTTCTTAACCTTAA |  |  |  |
| PEG3 | F:GGTGTAGAAGTTTGGGTAGTTG | 59 | 153 | chr19:57351945-57352097 |
|  | R: CTCACCTCACCTCAATACTAC  (5’ biotinylated) |  |  |  |

b. Pyrosequencing sequencing primers

| **Imprinted Locus** | **Sequencing**  **Primer sequence (5' - 3')** | **Size (bp)** | **Number**  **of CpG sites examined** | **GRCh37/hg19 coordinates** |
| --- | --- | --- | --- | --- |
| *TND* | GTAATTTAGGTAGTTTTAT | 93 | 8 | chr6:144328953-144329045 |
| *GRB10* | AATTAAACAACAAAAAACCC | 89 | 6 | chr7:50850569+  50850657 |
| *NESPAS* | TAAACTAAAAACTCTCAAAT | 190 | 5 | chr20:57430016+  57430205 |
| *PEG3* | TGTTTATTTTGGGTTGGT | 101 | 11 | chr19:57351945-57352045 |
