# Supplementary material for: Genetic variation affecting DNA methylation and the human imprinting disorder, Beckwith-Wiedemann syndrome
Source: Clin Epigenetics. 2018 Aug 30;10:114. doi: 10.1186/s13148-018-0546-4 (PMC6117921; doi:10.1186/s13148-018-0546-4)
Supplement: Supplementary file 2 — Figure S1. DNMT1 sequence variants identified in BWS patients. Figure S2. Sequence traces of DNMT1 variants generated by site-directed mutagenesis. Figure S3. Expression of GFP-tagged DNMT1 proteins in HeLa cells. Figure S4. Schematic of the trapping assay adapted from Frauer and Leonhardt (2009). (PPTX 1525 kb) [file 13148_2018_546_MOESM2_ESM.pptx]

## Slide 1
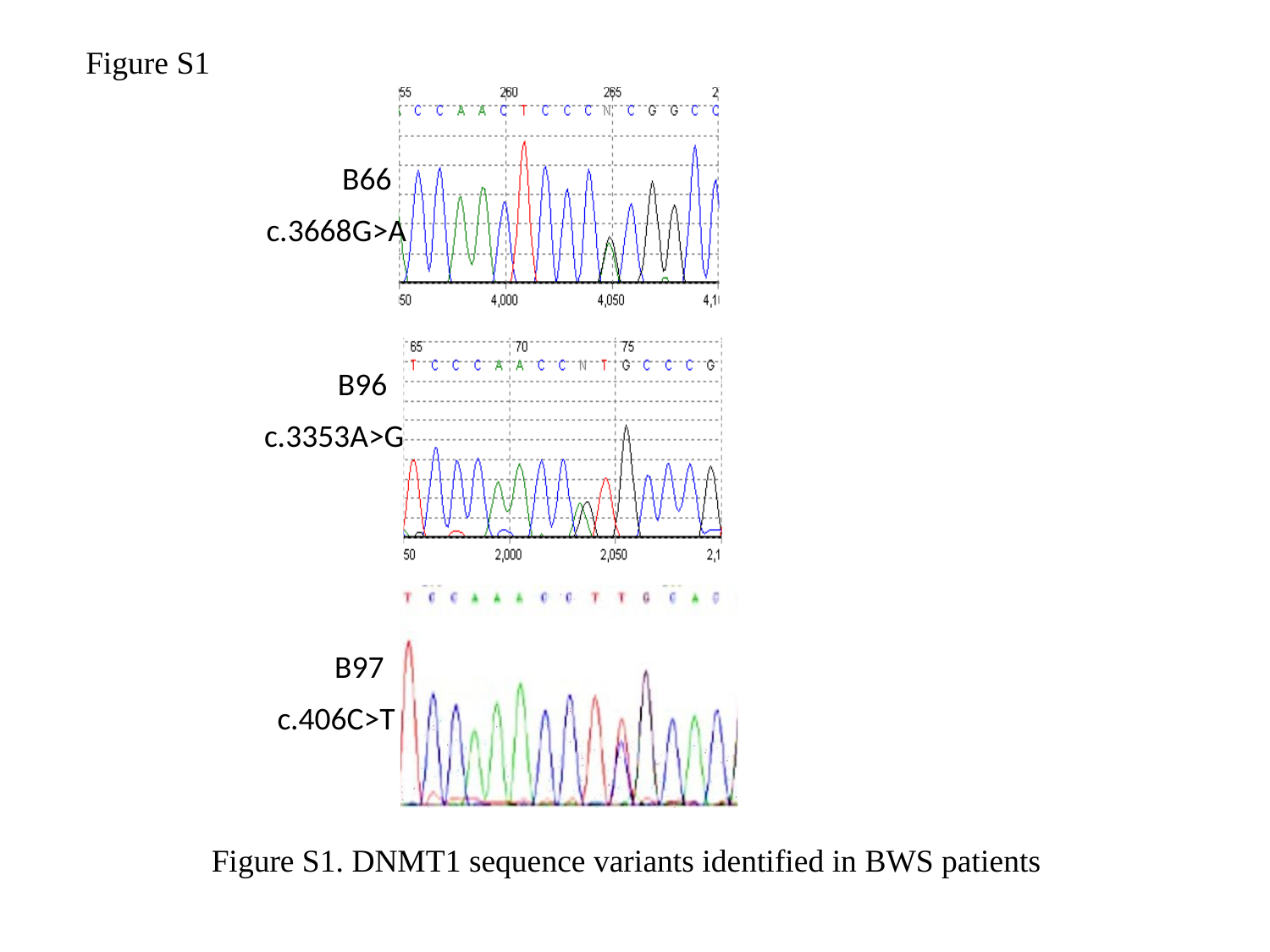

Figure S1
B66
c.3668G>A
B96
c.3353A>G
B97
c.406C>T
Figure S1. DNMT1 sequence variants identified in BWS patients

## Slide 2
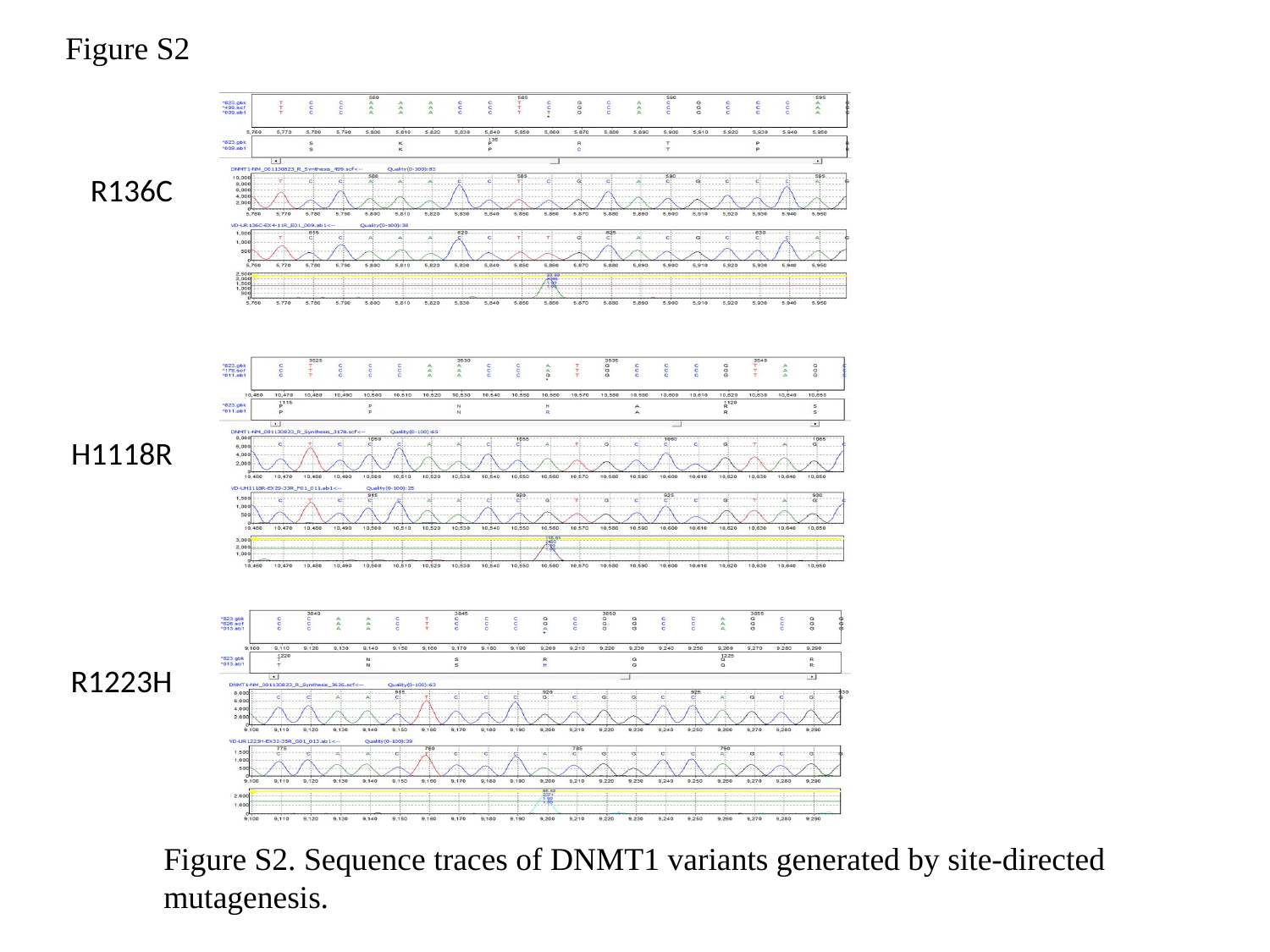

Figure S2
R136C
H1118R
R1223H
Figure S2. Sequence traces of DNMT1 variants generated by site-directed mutagenesis.

## Slide 3
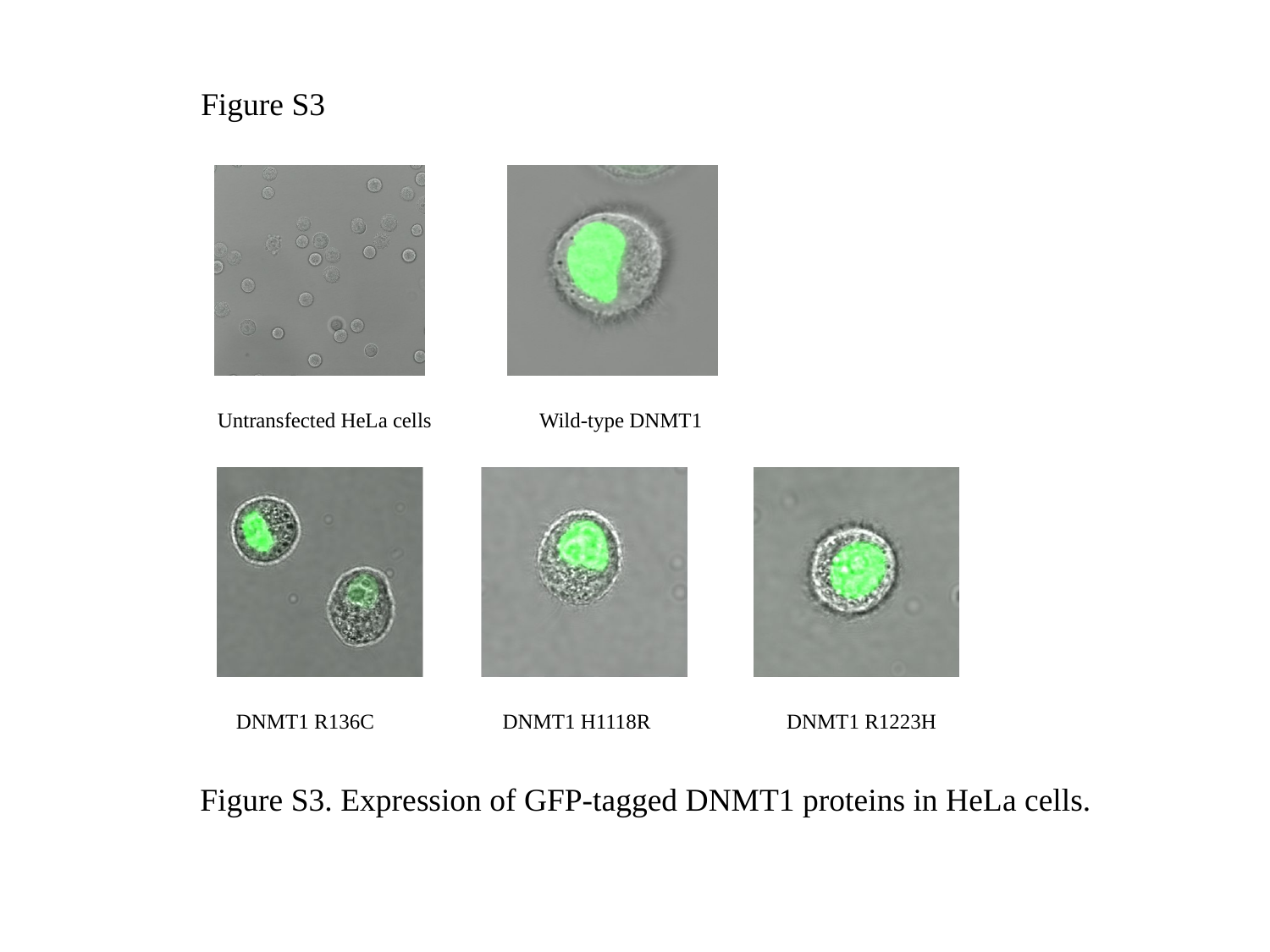

Figure S3
Untransfected HeLa cells
Wild-type DNMT1
 DNMT1 R136C
 DNMT1 H1118R
 DNMT1 R1223H
Figure S3. Expression of GFP-tagged DNMT1 proteins in HeLa cells.

## Slide 4
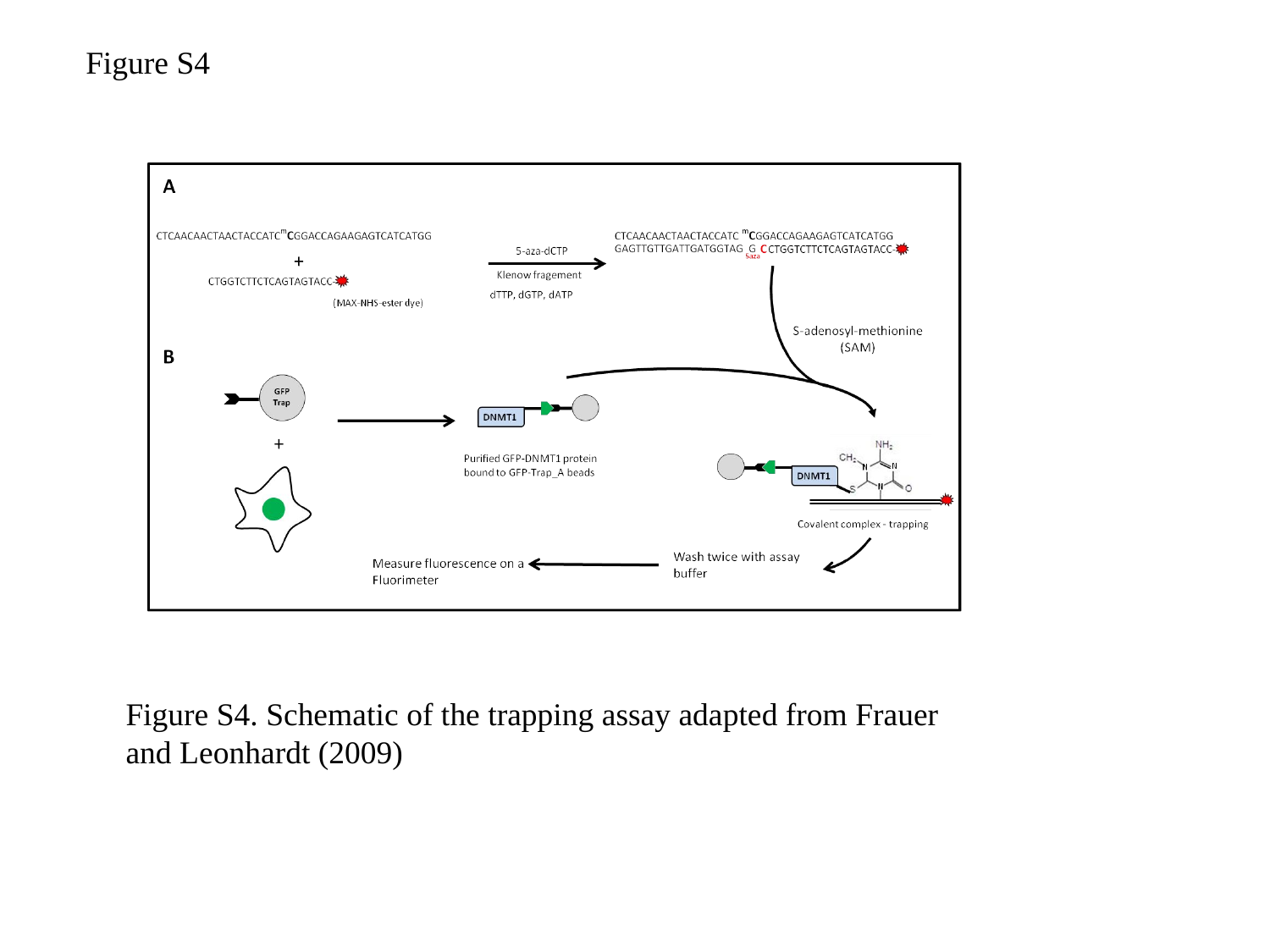

Figure S4
Figure S4. Schematic of the trapping assay adapted from Frauer
and Leonhardt (2009)
